# Microscopic Optical Projection Tomography *In Vivo*

**DOI:** 10.1371/journal.pone.0018963

**Published:** 2011-04-29

**Authors:** Matthias Rieckher, Udo Jochen Birk, Heiko Meyer, Jorge Ripoll, Nektarios Tavernarakis

**Affiliations:** 1 Institute of Molecular Biology and Biotechnology, Foundation for Research and Technology-Hellas, Crete, Greece; 2 Institute of Electronic Structure and Laser, Foundation for Research and Technology-Hellas, Crete, Greece; German Cancer Research Center, Germany

## Abstract

We describe a versatile optical projection tomography system for rapid three-dimensional imaging of microscopic specimens *in vivo*. Our tomographic setup eliminates the in xy and z strongly asymmetric resolution, resulting from optical sectioning in conventional confocal microscopy. It allows for robust, high resolution fluorescence as well as absorption imaging of live transparent invertebrate animals such as *C. elegans*. This system offers considerable advantages over currently available methods when imaging dynamic developmental processes and animal ageing; it permits monitoring of spatio-temporal gene expression and anatomical alterations with single-cell resolution, it utilizes both fluorescence and absorption as a source of contrast, and is easily adaptable for a range of small model organisms.

## Introduction

Imaging of cellular and molecular processes *in vivo*, as they transpire over time and in the context of the whole organism is becoming increasingly important in biomedical research. This capacity is of considerable relevance to studies of complex biological phenomena such as development and ageing. Currently available methodologies for imaging live small model organisms, such as *Drosophila* or *Caenorhabditis elegans* are based mainly on differential interference contrast (DIC) and confocal epi-fluorescence microscopy. While these approaches allow detailed two-dimensional (2D) visualization of cellular and sub-cellular structures, they are inefficient for obtaining high-resolution, three-dimensional (3D) reconstructions of whole animals over extended periods of time, during post embryonic development and ageing. In addition, confocal microscopy and the recently developed Single Plane Illumination Microscopy (SPIM) [1] are solely based on fluorescence detection and are not capable of imaging intrinsic or extrinsic absorption. Moreover, processing large numbers of individuals, which is required when studying stochastic processes such as ageing, is not practical with conventional DIC, confocal or SPIM microscopy.

Optical projection tomography (OPT), a relatively recently developed technology for 3D imaging, has emerged as a powerful tool for 3D reconstruction and visualization of specimen areas with sizes between 1–10 mm across [2], [3], [4], [5]. OPT is the optical equivalent of X-ray computed tomography (CT) scanning, where images of the attenuation coefficient or the emission distribution are obtained by applying an inverse Radon transform to back-propagate photons [6]. Initially, OPT was mostly used on fixed specimens after optical clearing in order to reduce photon scattering. The advent of Mesoscopic Fluorescence Tomography (MFT) and fluorescence mediated tomography (FMT) allowed efficient 3D imaging of live specimens, at mesoscopic or macroscopic scales [7], [8]. However, the absence of effective microscopic OPT implementations precluded the use of OPT *in vivo*, for imaging anatomical features of sub-millimeter-sized model organisms with adequate resolution. While such undersized specimens can, in principle, be processed by SPIM, this methodology is limited to fluorescence imaging, and cannot utilize absorption to discern anatomical features. Here, we describe a tomographic setup which addresses the challenge of microscopic OPT and allows rapid and high-resolution imaging of whole specimens *in vivo*.

## Results and Discussion

**Figure 1:**
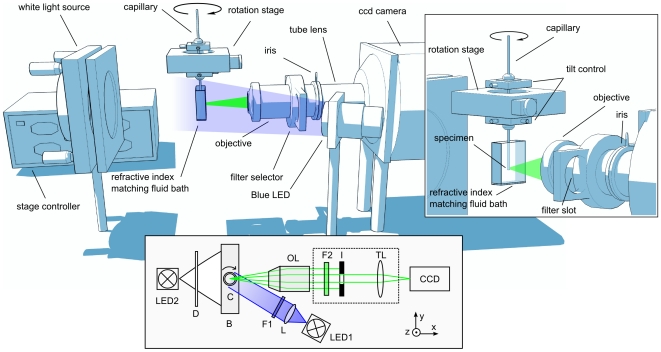
Overview of the experimental optical projection tomography system for *in vivo* imaging. This includes the schematic indicating all components of the OPT setup, including the rotation stage with capillary (C) placed in a refractive index matching bath (B), the two light sources (LED1+2), the objective (OL) and tube lens (TL) assembly featuring an emission filter (F2) and an iris diaphragm (I), and the CCD camera. The fluorescence excitation light (LED1) is roughly collimated (L) and its spectrum narrowed by an excitation filter (F1), whereas the white light (LED2) for trans-illumination passes a diffusor (D). A detailed close-up of the rotation stage and capillary is shown in the inset. The tilt screws on the rotation stage allow fine adjustment of the orientation and focusing of the sample. In this study, a white (LED2) or a blue (LED1) light source is used for sample illumination. Depending on the fluorophores used, appropriate light sources (and filters) can be easily interchanged. The detection system has a numerical aperture of NA = 0.22. A detailed description of the system is provided in the **Materials and Methods** section (see **Figure S1** for a photograph of the actual setup).

The individual components of the system and their arrangement are outlined in **Figure 1** (a photograph of the complete system is shown in **Figure S1a–c**). Nematodes are first immobilized in either the anesthetics levamisole or sodium azide, then transferred to halocarbon oil and finally mounted in a thin glass capillary, which is immersed in a chamber containing refractive index-matching fluid (see **Materials and Methods**). The capillary is attached to a rotation stage that allows precise positioning of the specimen for recording images from equidistant angles. To center the sample, the whole stage can be moved in all three dimensions through the stage controller, which in turn is controlled by customized software running on a computer (see **Materials and Methods**). Micrometric adjustment of the orientation and focusing of the specimen is facilitated by tilt controls on the rotation stage. The mounting stage is designed for easy coupling to microfluidics platforms, which have been developed for efficient manipulation and immobilization of nematodes [9], [10]. Thus, processing of large numbers of individuals with negligible specimen perturbation can be attained. These features are particularly important when monitoring stochastic phenomena, where large animal populations are considered, as in the case of ageing studies [11], or when probabilistic and quantitative phenotypic traits are involved, as in spatio-temporal analysis of gene expression and comparative anatomy.

Two separate light sources are used for illumination of specimens. A white light LED source provides trans-illumination for absorption imaging. For fluorescence imaging, a range of different wave length-emitting LEDs can be utilized for epi-illumination, depending on the fluorophores to be excited in the specimen. Thus, the system can be readily adapted for imaging existing fluorescent marker proteins as well as endogenous fluorophores (autofluorescence) and fluorescent dyes used for staining various cellular components, both *in vivo* and after fixation of the specimens. Furthermore, several chromophores can be specifically imaged simultaneously, in addition to the fluorescence and anatomy (white light) modalities by using appropriate filter combinations in the trans-illumination light path.The OPT setup allows image registration at video rate, and is capable of measuring 500 projections in fewer than 5 minutes, dependent on intensity of the fluorescent signal. The white light data can be acquired in 1.5 min, including 500 projection images over 360°.

**Figure 2:**
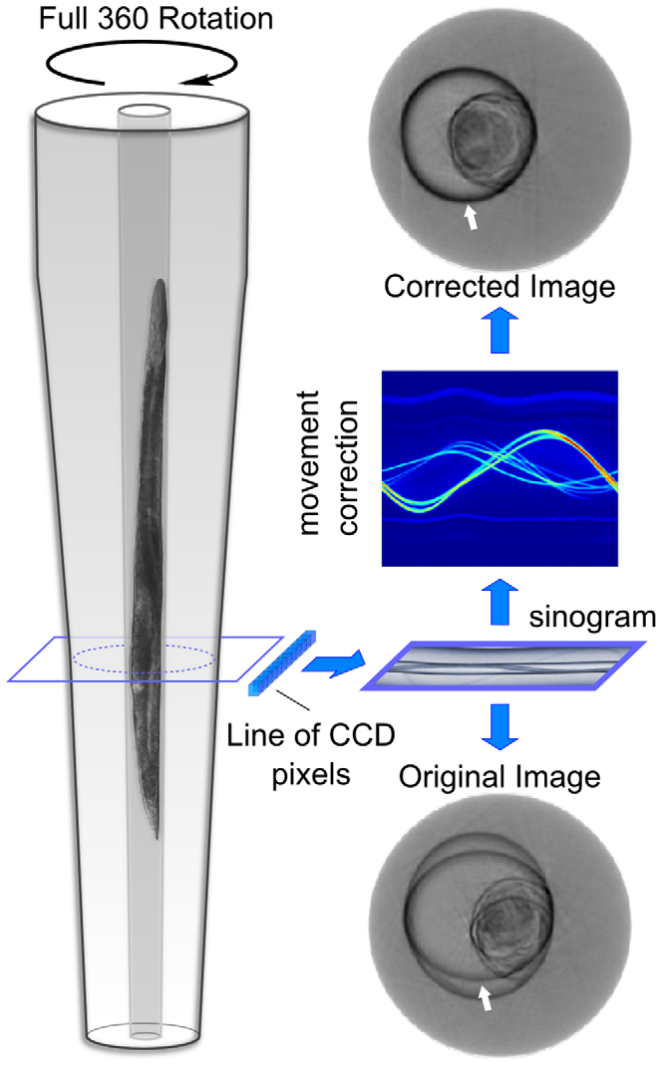
Delineation of image processing steps. A sinogram is obtained for each line of CCD pixels and all 360° measurements. After processing this sinogram to correct for movements of the specimen or drifts introduced by the experimental setup, the final adjusted image of a specimen slice is generated. The volumetric representation of the sample is obtained by stacking sequential slices. White arrows indicate the position of the inner wall of the capillary.

A major challenge, which has hindered the use of OPT for microscopic imaging *in vivo*, is the residual random movement and drift of the sample during the course of observation, which becomes increasingly significant at higher magnifications required for microscopic OPT [12]. By using landmarks in the organism, which are visible in all projection images, we correct for lateral movements of the sample. Additionally, we developed software, which compensates for slight non-circular motion of the rotation stage, by detecting changes in the predicted trajectories of the images consisting of data from a single line of CCD pixels for all angular measurements (sinograms) [13]. Both correction algorithms are applied prior to data reconstruction (see **Figure 2**, **Materials and Methods**). Filtered back projections of specimen images acquired at a number of equidistant angles are then used for 3D reconstruction. This processing enables visualizations of individual cells and cellular structures at high 3D resolution of about 2 µm, not previously possible with OPT.

**Figure 3:**
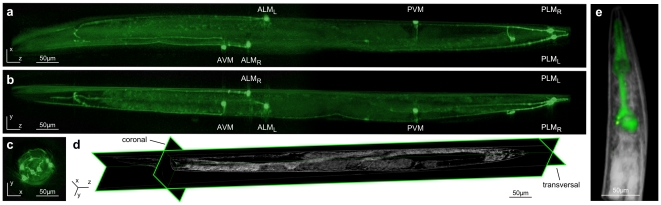
Tomographic reconstruction of wild type and transgenic *C. elegans* allows detailed analysis of anatomical features and fluorescence expression patterns. (**a–c**) Maximum intensity projections of a transgenic animal expressing GFP in mechanosensory neurons (see also **Video S1**). The three viewpoints, sagittal (**a**), transversal (**b**) and coronal (**c**) reveal the circuit of the 6 labeled mechanosensory neurons (ALM_L/R_, AVM, PLM_L/R_, PVM). (**d**) Assembled single slices of a 3D reconstruction of brightfield data, displaying the anatomy of *C. elegans*. Combined sagittal, transversal and coronal views allow visualization of structures such as intestine, pharynx, gonad and eggs. (**e**) Merged, brightfield and fluorescence tomographic reconstruction of the anterior part of *C. elegans*. Pharyngeal muscles expressing GFP are shown in green (see also **Video S2**). Size bar indicates 50 µm.

For experimental verification of the capacity for *in vivo*, high-resolution imaging by microscopic OPT, we used the in-house built tomographic system shown in **Figure S1** to image live transgenic *C. elegans* animals, carrying a green fluorescent protein (GFP) reporter fusion expressed specifically in only the 6 mechanosensory neurons of the nematode [14]. These neurons extend processes that, in two sets of three, span the length of the animal (**Figure 3a–c**, **Video S1**). The three viewpoints shown in panels **a–c** (sagittal, transversal and coronal respectively) are depicted in **Figure 3d**, for the corresponding white light data. Notwithstanding their diameter of approximately 2.7 µm, mechanosensory neuron processes are clearly visible, demonstrating the capacity of the system for highly sensitive 3D reconstruction at the level of living single cells, within the context of the whole organism. Such fine cellular features and anatomical details are not visible without sample movement correction, implemented on sinograms before reconstruction. Visualization and monitoring of alterations in individual neuronal axons is essential in the dissection of neuron degeneration and regeneration, two processes which are extensively studied in *C. elegans* [15], [16].

**Figure 4:**
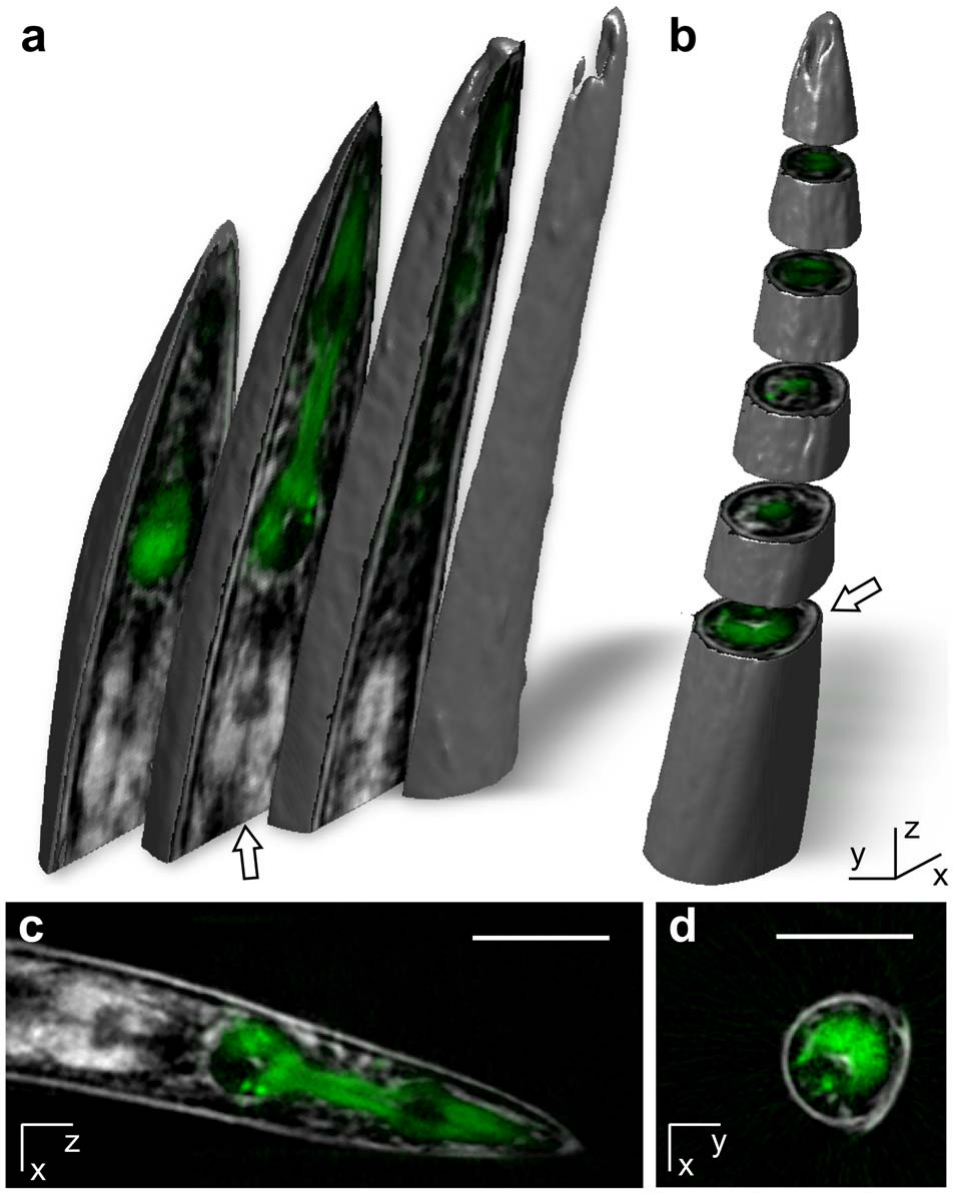
3D rendering of the anterior part of the *C. elegans* body. (**a**, **b**) 3D visualizations of anterior body anatomy (see also **Video S3**). Three transversal and five coronal sections are shown in (**a**) and (**b**), respectively. The 3D view allows detailed visualization and analysis of anatomical features, down to single cell level (see also **Video S1**, **S4**). The process for generating 3D renderings is described in the **Materials and Methods** section. (**c**, **d**) Two-dimensional, single slices in transversal and coronal planes, respectively, are shown (indicated by white arrows in (**a**) and (**b**) respectively). In all four panels, brightfield and fluorescence images are merged. Pharyngeal muscle cells expressing GFP are shown in green (see also **Video S2**). Size bar indicates 50 µm.

Reconstructed data derived from both absorption and fluorescence images can be combined to obtain anatomical information about gene expression patterns. This is illustrated in **Figure 4**, which shows a merged brightfield and fluorescence reconstruction of pharyngeal muscle cells expressing GFP in the anterior part of the *C. elegans* body (a full 3D reconstruction is shown in **Video S2**). In addition, as shown in **Figure 4**, accurate, *in silico*, 2D sectioning of the specimen as well as for volumetric 3D rendering of specific parts or of the entire animal is possible in this manner (**Video S3**). Coronal, sagittal and transversal sections through the specimen allow retrieval of precise anatomical information down to single cell level (**Video S4**). The potential for high-resolution 3D fluorescence imaging coupled with absorption/brightfield-derived anatomical information is critical for analysis of gene expression using fluorescent reporter proteins, in addition to studies of bio-molecule co-localization in the context of the whole organism, *in vivo*. Moreover, the tomographic setup described here permits fast acquisition of imaging data, which in turn facilitates time-lapse OPT for spatio-temporal representation of changes in morphology, cell positioning, gene expression levels and molecular movement over extended periods of time, *in vivo* and through the entire animal (4D microscopy/imaging). This is becoming increasingly important for studies of development and ageing. Currently available conventional microscopy methodologies either do not allow, or are not optimized for such applications.

In summary, we present a simple and cost effective tomographic setup, which achieves fast 3D imaging of live *C. elegans* animals at single-cell resolution. The system is highly versatile and configurable for both absorption/brightfield and fluorescence imaging, with multiple chromophores and fluorophores. By post-acquisition filtering of residual sample movement and spurious drifts we have accomplished visualization of neuronal features, such as single axons and dendrites *in vivo*. This is not possible with other, previously described OPT implementations. Thus, the system is particularly suited for following neuron and axonal migration during development, as well as neurodegeneration, regeneration and other dynamic phenomena, at microscopic scale. We also note that the system can be adapted for other small model organisms such as *Drosophila*, other invertebrates, and small marine crustaceans, which have emerged as powerful models for evo-devo studies. Finally, outfitting for specimen handling using microfluidics should facilitate high throughput OPT of large cohorts of live animals which, we anticipate, would greatly benefit studies of intrinsically stochastic biological phenomena such as ageing.

## Materials and Methods

### Sample preparation

We used standard methods for *C. elegans* maintenance, crosses and other genetic manipulations [17]. Nematodes were cultured at 20°C. The *Escherichia coli* strain OP50 was used as a food resource. The following strains and transgenic animals were used in this study: N2: wild-type Bristol isolate, *Is* [p*_mec-4_*GFP], expressing GFP in the six mechanosensory neurons; *Ex* [p*_myo-2_*GFP], expressing GFP in the pharynx. Glass capillaries (5 µl) were modified using a capillary puller (PN-30, Narishige, Tokyo, Japan). The tip was tapered to reduce the inner diameter for enhanced sample stability and positioning. Young adult worms can be immobilized in a 5–10 mM levamisole (Sigma-Aldrich, St. Louis, USA), or in a 10 mM sodium azide (NaN_3_, Sigma-Aldrich, St. Louis, USA) solution for 15 min at 20°C and then transferred in a drop of halocarbon oil (refractive index n = 1.42). Levamisole temporarily suppresses muscular activity by blocking the function of nicotinic acetylcholine receptors (nAChR), while NaN_3_ inhibits both cytochrome *c* oxidase and adenosine triphosphate (ATP) synthase. In the applied concentration and incubation time these drugs are not lethal for *C. elegans*. Residual anaesthetic was removed by moving the worm in the oil with a hairpin. Animals were sucked into the tip of the capillary by a pipette. The capillary was carefully cleaned with 70% ethanol and then sealed at both ends by slightly dipping into liquid glue (Glue-All, Multi-Purpose Glue, Elmer's Products, OH, USA).

### Tomographic setup

The light source comprises superbright LEDs (Luxeon V Star 5 W, Philips Lumileds Lighting, San Jose, CA, USA) in numerous colors, allowing excitation of most commercially available fluorescent dyes. White LEDs are used for transmission (absorption) imaging. Although, for this study blue LEDs were employed for excitation due to the use of GFP, the system is not restricted, allowing any chromophore or fluorescent protein can be visualized by using appropriate light sources. The sample holder is based on a rotation system, containing a high resolution rotation stage (8MR180, Standa, Vilnius, Lithuania) with 36000 steps per revolution. Attached is a custom made capillary holder, holding standard single use micro-capillaries (Blaubrand® - intraMARK, BRAND GmbH, Wertheim, Germany). Capillaries are immersed in a custom-made refractive index matching vessel, assembled of 50×24×0.15 mm borosilicate cover slips (n_w_ = 1.474), containing 87% glycerol solution (Sigma-Aldrich, St. Louis, MO, USA) as a refractive index matching fluid (n_f_ = 1.474) to minimize internal reflections and refraction of the excitation and emission light. The imaging unit consists of a lens tube system (InfiniTube™, Infinity, Boulder CO, USA) attached to a custom-made filter slide, which can hold up to three 25 mm diameter fluorescence filters (in this particular study, we used a 525±17.5 nm band-pass filter for GFP, 593±20 nm for DsRed, both Semrock®, Rochester NY., USA). In this study, we used a 10× infinity corrected microscope objective lens (Mitutoyo, Kawasaki, Japan). The objective lens has a numerical aperture NA of 0.28, which is reduced to typically around 0.22 by the iris diaphragm. The NA for trans-illumination is <0.035, for fluorescence illumination it is ca. 0.1. Except for the collimation lens L, all lenses are corrected for chromatic aberrations. The detection filter F2 and the iris are placed next to the back focal plane of the objective. Depending on the specimen size, infinity corrected lenses with a range of other magnifications are available and can also be used. The objective lens is attached to a thermoelectrically cooled, electron multiplying CCD with 1002×1004 pixels (Ixon DV885, ANDOR™ Technology, Belfast, Northern Ireland). To increase the focal depth of the system, a variable iris is placed behind the objective, as described previously [18]. The tilt of the sample is corrected mechanically prior to the experiment, while drifts and sample movements are corrected by applying algorithms during post-processing. A schematic of the system is shown in **Figure 1** and the actual setup is depicted in **Figure S1**).

### Data acquisition and 3D reconstruction

Data was acquired using custom software based on LabView™ to control specimen positioning, the camera and the rotation stage. The software made use of specific functions developed in C++ for speed improvement. The experiment graphical user interface (GUI) is capable of changing all experimental parameters (camera, stage movement, and filters). 500 images of the rotating specimen were taken from equidistant angles (in 0.72° steps) and stored as one collected file with a size of approximately one gigabyte. The software is developed in MATLAB (http://www.mathworks.com/) and includes a user-friendly interface and a detailed how-to manual. The full software package is available under the GNU General Public License (GPL; http://www.gnu.org/licenses/gpl.html) and can be downloaded at the following address: http://elegans.imbb.forth.gr/opt/ Bright light image data was obtained by back-illumination with a white light LED. A separate set of fluorescence data was recorded under epi-illumination with a blue light LED, which was selected in accordance to the fluorophore used in this study (GFP). An in-house developed toolbox of custom MatLab evaluation scripts was used to analyze the 3D data stacks containing the projection views on a PC (2.8 GHz, 4 GB RAM). To account for movement and motility of the live specimen and also for minor non-circular motion of the rotation stage, we developed software which considers changes in the predicted trajectories of the sinograms (images consisting of data from a single line of CCD pixels for all angular measurements) and corrects for these prior to data reconstruction [19]. Correction along the rotation axis for the data shown here took only a couple of seconds, while the iterative correction took about 1 h. Resolution is significantly degraded if this approach is not used, indicating its importance when imaging *in vivo* biological material with OPT. Once raw data were processed to correct for movement and drifts, in both fluorescence and white light mode, 3D reconstructions were calculated by sequentially stacking 2D slices reconstructed using a filtered back-projection algorithm with a Hann-Filter implemented together with an inverse Radon-transform [20].

## Supporting Information

Figure S1 **Photographs of the OPT setup indicating all components of the system.** (**a**) The complete experimental setup. (**b**, **c**) Main components of the system, including the rotation stage and sample capillary. The inset in (**b**) is a magnified view of a single specimen image taken from one angle. 500 such raw data images are recorded from equidistant angles and are later post-processed for 3D reconstruction. (**c**) A close-up of the rotation stage and the refractive index matching fluid container is shown. In the instance shown, the blue LED light source is activated.
(TIF)

Video S1 **Neuroanatomy of nematode mechanosensory neurons.** 3D reconstruction of the six GFP-labeled touch receptor neurons in the context of the whole animal.
(MOV)

Video S2 **Merged reconstruction of GFP-expressing pharyngeal cells and anatomical features of the anterior part of** ***C. elegans*** **.**
(MOV)

Video S3 **A scan through the reconstruction of a bright field recording of the anterior part of** ***C. elegans*** **.** Blue-green rendering emphasizes anatomical structures (pharynx, intestine).
(MOV)

Video S4 **Combined bright field and fluorescent 3D renderings facilitate analyses of gene expression patterns.** As an example, the GFP-labeled touch receptor neurons and the pharynx in the anterior part of the animal are shown.
(MOV)

## References

[1] Huisken J, Swoger J, Del Bene F, Wittbrodt J, Stelzer EH (2004). Optical sectioning deep inside live embryos by selective plane illumination microscopy.. Science.

[2] Boot MJ, Westerberg CH, Sanz-Ezquerro J, Cotterell J, Schweitzer R (2008). In vitro whole-organ imaging: 4D quantification of growing mouse limb buds.. Nat Methods.

[3] Sharpe J, Ahlgren U, Perry P, Hill B, Ross A (2002). Optical projection tomography as a tool for 3D microscopy and gene expression studies.. Science.

[4] Colas JF, Sharpe J (2009). Live optical projection tomography.. Organogenesis.

[5] McGinty J, Tahir KB, Laine R, Talbot CB, Dunsby C (2008). Fluorescence lifetime optical projection tomography.. J Biophotonics.

[6] Kak A, Slaney M (1988). Principles of Computerized tomographic imaging.

[7] Ntziachristos V, Tung CH, Bremer C, Weissleder R (2002). Fluorescence molecular tomography resolves protease activity in vivo.. Nat Med.

[8] Vinegoni C, Pitsouli C, Razansky D, Perrimon N, Ntziachristos V (2008). In vivo imaging of Drosophila melanogaster pupae with mesoscopic fluorescence tomography.. Nat Methods.

[9] Chronis N, Zimmer M, Bargmann CI (2007). Microfluidics for in vivo imaging of neuronal and behavioral activity in Caenorhabditis elegans.. Nat Methods.

[10] Ben-Yakar A, Chronis N, Lu H (2009). Microfluidics for the analysis of behavior, nerve regeneration, and neural cell biology in C. elegans.. Curr Opin Neurobiol.

[11] Artal-Sanz M, Tavernarakis N (2009). Prohibitin couples diapause signalling to mitochondrial metabolism during ageing in C. elegans.. Nature.

[12] Miao Q, Rahn JR, Tourovskaia A, Meyer MG, Neumann T (2009). Dual-modal three-dimensional imaging of single cells with isometric high resolution using an optical projection tomography microscope.. J Biomed Opt.

[13] Birk UJ, Rieckher M, Konstantinides N, Darrell A, Sarasa-Renedo A Correction for specimen movement and rotation errors for in-vivo Optical Projection Tomography.. Biomed Opt Express.

[14] Chalfie M, Tu Y, Euskirchen G, Ward WW, Prasher DC (1994). Green fluorescent protein as a marker for gene expression.. Science.

[15] Syntichaki P, Tavernarakis N (2003). The biochemistry of neuronal necrosis: rogue biology?. Nat Rev Neurosci.

[16] Guo SX, Bourgeois F, Chokshi T, Durr NJ, Hilliard MA (2008). Femtosecond laser nanoaxotomy lab-on-a-chip for in vivo nerve regeneration studies.. Nat Methods.

[17] Brenner S (1974). The genetics of Caenorhabditis elegans.. Genetics.

[18] Ripoll J, Ntziachristos V (2004). Imaging Scattering media from a distance: theory and applications of non-contact optical tomography.. Modern Physics Letters B.

[19] Katsevich A (2010). An accurate approximate algorithm for motion compensation in two-dimensional tomography.. Inverse Problems.

[20] Kak A, Slaney M (2001). Principles of computerized tomographic imaging..

