# Supplementary figures and images for: Microscopic Optical Projection Tomography In Vivo
Source: PLoS One. 2011 Apr 29;6(4):e18963. doi: 10.1371/journal.pone.0018963 (PMC3084718; doi:10.1371/journal.pone.0018963)

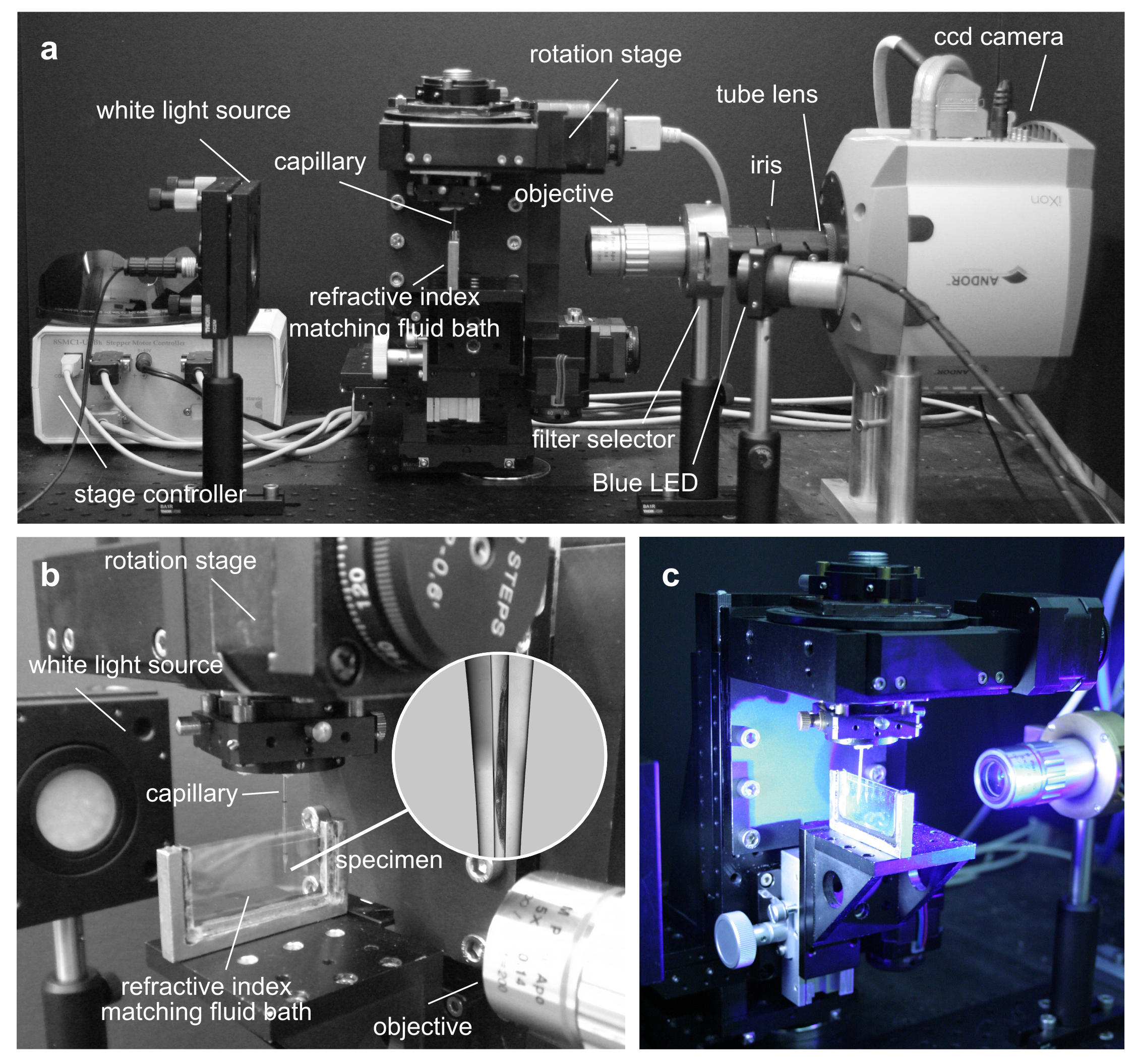

Supplement: Figure S1 — Photographs of the OPT setup indicating all components of the system. (a) The complete experimental setup. (b, c) Main components of the system, including the rotation stage and sample capillary. The inset in (b) is a magnified view of a single specimen image taken from one angle. 500 such raw data images are recorded from equidistant angles and are later post-processed for 3D reconstruction. (c) A close-up of the rotation stage and the refractive index matching fluid container is shown. In the instance shown, the blue LED light source is activated. (TIF) [file pone.0018963.s001.tif]
